# Ruxolitinib synergizes with regulatory T cells to improve inflammation but has no added benefits in decreasing albuminuria in SLE

**DOI:** 10.3389/fimmu.2025.1449693

**Published:** 2025-02-05

**Authors:** Mi-Ae Lyu, Ximing Tang, Maria Gabriela Raso, Meixian Huang, Ke Zeng, Tara Sadeghi, Christopher R. Flowers, Simrit Parmar

**Affiliations:** ^1^ Department of Lymphoma/Myeloma, The University of Texas M.D. Anderson Cancer Center, Houston, TX, United States; ^2^ Department of Translational Molecular Pathology, The University of Texas M.D. Anderson Cancer Center, Houston, TX, United States; ^3^ Cellenkos Inc., Houston, TX, United States; ^4^ Department of Microbial Pathogenesis & Immunology, Texas A&M University, Bryan, TX, United States

**Keywords:** adoptive cell therapy, regulatory T cells (Tregs), allogeneic, umbilical cord blood (UCB), ruxolitinib, JAK/STAT pathway, inflammation, systemic lupus erythematosus (SLE)

## Abstract

**Background:**

Umbilical cord blood (UCB)-derived CD4^+^CD25^+^CD127^low^ regulatory T cells (Tregs) can decrease albuminuria and anti-dsDNA IgG in systemic lupus erythematosus (SLE). Ruxolitinib, a JAK/STAT inhibitor, has been shown to improve cutaneous manifestations of SLE. We hypothesize that the addition of ruxolitinib to UCB-Tregs may improve SLE outcomes.

**Methods:**

*In vitro* cell suppression, phenotype change, IL-10 secretion, and cytokine levels in coculture supernatants were determined to quantify the impact of adding ruxolitinib to UCB-Tregs. A xenogeneic SLE model was utilized to study their *in vivo* combination.

**Results:**

In a dose-dependent manner, ruxolitinib addition synergizes with UCB-Tregs to suppress SLE-PBMC proliferation, inhibit CD8^+^ T cells, and reduce phosphorylation of STAT3/STAT5/AKT in CD8^+^ T cells. UCB-Treg and ruxolitinib combination also downregulates the soluble form of inflammatory cytokines including IFN-γ, IP-10, TNF-α, IL-6, sCD40L, IL-17A, IL-17F, IL-1α, and LIF in cocultures. The addition of ruxolitinib increases UCB-Treg cell persistence in peripheral blood *in vivo* and decreases the soluble form of human inflammatory cytokines including IFN-γ, TNF-α, and sCD40L in plasma along with improvement of skin lesions in SLE xenografts. Compared to control, significantly lesser CD3^+^, CD4^+^, CD8^+^, and Ki-67^+^ infiltrates are observed in the lung and kidney of UCB-Tregs and/or ruxolitinib recipients. No added benefit of addition of ruxolitinib is observed on the significant improvement in the urine albumin/creatinine ratio and the anti-dsDNA IgG levels induced by UCB-Tregs.

**Conclusions:**

Our results demonstrate that the addition of ruxolitinib to UCB-Tregs increases UCB-Tregs suppressor function and their persistence *in vivo*, downregulates systemic inflammation, and controls cutaneous SLE but does not add to UCB-Treg-mediated improvement in renal manifestations.

## Highlights

- *In vitro*, the addition of ruxolitinib to UCB-Tregs augments the inhibition of pathogenic CD8^+^ T cells, and the reduction of soluble IFN-γ, IP-10, TNF-α, IL-6, sCD40L, IL-17A, IL-17F, IL-1α, and LIF production.
- *In vivo*, ruxolitinib improves UCB-Treg cell persistence and decreases CD3^+^, CD4^+^ T, CD8^+^ T, and Ki-67^+^ cell tissue infiltration but has no added benefits to albuminuria and anti-dsDNA IgG Ab.

## Introduction

Although traditionally thought to be a B-cell disorder, recent data support a central role of T cells in the pathogenesis of systemic lupus erythematosus (SLE) (1). T cell dysregulation and autoreactive T effector (Teff) cells affect peripheral tolerance and induce inappropriate activation of B cells (2). T lymphocytes are increasingly being recognized as key contributors to disease pathogenesis, where CD4 T follicular helper cells enable autoantibody production, inflammatory Th17 subsets promote inflammation, while defects in regulatory T cells (Tregs) lead to unchecked immune responses (3).

Th1 cytokines including IFN-γ promote B-cell class switching and stimulate pathogenic autoantibody production in SLE (4). Levels of IFN-γ are elevated in patients with SLE compared to controls and positively correlate with SLE disease activity index (SLEDAI) scores (5). Th2 cytokines including IL-4 promote B-cell differentiation into plasma cells and induce antibody class switching to IgG1 and IgE (6). In lupus-prone mice, blocking IL-4 decreases anti-double-stranded DNA (anti-dsDNA) antibodies, whereas the administration of IL-4 increases the levels of this autoantibody (7). Th17 cytokines have been shown to correlate with the SLE severity where IL-17 levels correlate with SLEDAI scores, and both IL-17 and IL-23 are associated with treatment-resistant active nephritis (8, 9). Therefore, autoreactive T cells drive the severity of disease in SLE (2).

Tregs develop in the thymus through strong T cell receptor (TCR) signaling just below the threshold for negative selection and recognize self-antigens for their differentiation (10). Tregs express the CD4^+^CD25^+^CD127^low^ phenotype with high intracellular FOXP3 (11). Autoreactive T cells that escape negative selection in the thymus can persist in the periphery where Tregs prevent their aberrant activation and expansion, thus acting as important gatekeepers (12). By neutralizing these autoreactive T cells as well as other harmful immune cells, Tregs play a central role in the resolution of unwanted inflammation (11, 13). Furthermore, Tregs homing to the areas of inflammation are based on their ability to sense the source of survival cytokine, IL-2, and migration to the zones of immune activation where they “steal” IL-2 from effector T cells promoting their apoptosis (14). In all, Tregs perform several functions including (i) preventing autoimmunity, (ii) suppressing inflammation, (iii) maintaining tissue integrity, (iv) controlling allergies including asthma, (v) inducing tolerance to dietary antigens and the fetus, and (vi) protecting commensal bacteria from being eliminated by the immune system (15). Defective and decreased Tregs can lead to autoimmune diseases including SLE (10). In fact, compared to a healthy population, SLE patients have been shown to have a lower percentage of Tregs and have defects in their suppressor function (16–19). Furthermore, the pathogenic Teff cells in SLE patients develop resistance to Treg-induced suppression that supports their unopposed proliferation, leading to excessive secretion of inflammatory cytokines that contribute to SLE pathogenesis (20).

Recently, we showed that umbilical cord blood (UCB)-derived Treg cells (UCB-Tregs) have unique properties including (i) lack of plasticity when exposed to inflammatory micro-environments; (ii) no requirement for HLA matching with the recipients; (iii) long shelf life of the cryopreserved cells; and (iv) immediate product availability for on-demand treatment (21). In addition, UCB-Tregs are able to decrease the production levels of anti-dsDNA IgG antibody and inflammatory cytokines and improve albuminuria in the xenogeneic model of SLE (22). In the same study, it was shown that UCB-Tregs are able to suppress pathogenic SLE PBMCs to a similar extent as the healthy PBMCs (22). Therefore, adoptive therapy with UCB-Tregs for SLE might be a promising approach. A concern remains for the possibility of resistance since in response to the unusually high levels of plasma cell-niche cytokines in SLE patients, the auto-antibody production is dependent on JAK/STAT3 activation such that this process can be abrogated by inhibition of this pathway by the JAK inhibitor, ruxolitinib (23). Although ruxolitinib as a single agent has shown significant activity in steroid refractory acute graft vs. host disease (GVHD), a T cell-mediated fatal complication of allogeneic stem cell transplantation (24), when used as a therapeutic strategy for SLE, despite showing activity in cutaneous lupus by decreasing inflammation, no improvement in the systemic organ involvement was observed in ruxolitinib recipients (25–27). The combination of ruxolitinib with Tregs has been examined in a GVHD model with an observed decrease in the incidence and severity and improved survival without dampening of the graft versus leukemia effect (28). We have shown a synergistic effect of UCB-Tregs and ruxolitinib in xenogeneic GVHD as well (29).

Here, we hypothesize that the combination of ruxolitinib with UCB-Tregs may lead to a synergistic activity in treating SLE manifestations.

## Materials and methods

### Cell source

UCB-Treg cells were generated as described previously (21). Human peripheral blood mononuclear cells from SLE patients (SLE-PBMCs, BioIVT, Westbury, NY, USA) or healthy donors (HD-PBMCs, Gulf Coast Blood Bank, Houston, TX, USA) and UCB-Tregs were cultured as described previously (22). Additionally, cryopreserved UCB-derived *ex vivo* expanded CD4^+^CD25^+^127^low^ Treg cells were obtained from Cellenkos Inc. (Houston, TX, USA).

### Phenotype analysis

UCB-Treg cells, SLE-PBMCs, or UCB-Treg: SLE-PBMC (1:1) cocultures were stained using the following antibodies: APC-eFluor 780-CD45 (HI30), Alexa Fluor 532-CD3 (UCHT1), FITC-CD3 (UCHT1), PerCP-Cyanine5.5-CD8a (RTA-T8), Super Bright 600-CD19 (SJ25C1), PE-CD25 (BC96), PE-Cy5-CD127 (eBioRDR5), APC-CD56 (CMSSB), FITC-CD16 (eBioCB16) (CB16), PerCP-eFluor 710 CD14 (61D3), PE-Cy7-HLA-DR (LN3), PE-Cyanine7-phosphor-STAT3 (Tyr705) (LUVNKLA), and LIVE/DEAD™ fixable Blue (Thermo Fisher Scientific); BV650-CD4 (L200), BV510-CD8 (RPA-T8), PE-CF594-CD27 (M-T271), Alexa Fluor 700-IgD (IA6-2), BV421-CD62L (SK11), Alexa Fluor 647-Helios (22F6), Alexa Fluor 647-FoxP3 (259D/C7), PerCP-Cy5.5-FoxP3 (236A/E7), Pacific Blue-Stat5 (pY694) (47/Stat5) (pY694), BV421-Akt (pS473) (M89-61), and BUV-395-Ki-67 (B56) (BD Biosciences); and Pacific Blue-CD45.1 (A20) (Southern Biotech, Birmingham, AL, USA). T, Treg, B, NK cells, and monocytes were gated and displayed on the t-distributed stochastic neighbor embedding (t-SNE). The following CD8^+^ T cells were further analyzed: p-Stat3^+^, p-Stat5^+^, Ki-67^+^, or p-Akt^+^ CD8^+^ T cells. Cytek Aurora (Cytek Biosciences, Fremont, CA, USA), BD LSRFortessa (BD Biosciences), and FlowJo software (FlowJo, LLC, Ashland, OR, USA) were used for phenotype analysis.

### Ruxolitinib IC_50_

Ruxolitinib (INCB018424; S1378) was purchased from Selleck Chemicals (Houston, TX). The comparative half-maximal inhibitory concentration (IC_50_) values of ruxolitinib against HD-PBMCs (Gulf Coast Blood Bank), SLE-PBMCs (BioIVT), or UCB-Tregs after treatment with ruxolitinib for 6 days were assessed as described previously (30).

### Functional ability of UCB-Treg cells

In the presence of 0, 25, or 100 nmol/L ruxolitinib and CD3/CD28 beads, the suppressive function of UCB-Tregs on the proliferation of CD4^+^CD25^−^ conventional T cells (Tcons) or SLE-PBMCs were assessed as described previously (21, 22).

### Production levels of soluble human cytokines

UCB-Tregs, SLE-PBMCs, or UCB-Treg: SLE-PBMC (1:1) cocultures were cultured for 3 or 6 days in the absence or presence of ruxolitinib. The soluble form of human cytokines was measured using Cytokine ELISA kits from Thermo Fisher Scientific or Eve Technologies (Calgary, AB, Canada).

### SLE xenograft model

Animal procedures were performed according to an approved IACUC protocol by The University of Texas MD Anderson Cancer Center. *In vivo* efficacy assessment was done using a SLE xenograft model as described previously (21, 22). Mice were divided into four groups (No Rx, Rux, UCB-Treg, and UCB-Treg+Rux, *n* = 5 mice/group) after they displayed human immune cells. A total of 5 × 10^6^ UCB-Treg cells were administered by tail vein injection on days 28, 32, 42, and 46. Ruxolitinib was administered orally at a dose of 90 µg on days 35, 36, 37, 38, 39, 49, 50, 51, 52, and 53. The reconstitution level of human immune cells and weight loss was monitored weekly or twice per week, respectively. Mice were euthanized using 30%–70% displacement of the chamber volume per minute with compressed CO_2_, and death was confirmed by cervical dislocation. At the time of euthanasia, single-cell suspensions from each organ were aseptically isolated and phenotypic analysis of human immune cells was determined based on the expression levels of human cell surface and intracellular markers.

### Urinary albumin in SLE xenografts

For the kidney function assessment, the production levels of albumin and creatinine in mouse urine samples were assessed as described previously (22).

### Anti-human dsDNA IgG antibody and human cytokine/chemokine in SLE xenografts

The production levels of anti-human dsDNA IgG Ab and human inflammatory cytokines in mouse plasma samples were measured as described previously (22).

### Histopathology and immunohistochemistry

Mouse organs were harvested, fixed, processed, and embedded in paraffin. For histopathology analysis, tissue sections were stained with hematoxylin and eosin. The presence of human immune cells in the mouse tissues was assessed by immunohistochemical staining with the following antibodies: human CD3 (Clone F7.2.38) (DAKO, Santa Clara, CA, USA), CD4 (Clone 4B12) (Leica Biosystems Inc., Buffalo, Grove, IL, USA), CD8 (Clone C8/144B) (Thermo Fisher Scientific), and Ki-67 (Clone MIB-1) (DAKO). The stained tissue slides were scanned using Aperio AT2 (Leica Biosystems Inc., Buffalo Grove, IL, USA). Total positive cells and *H*-scores for human CD3, CD4, CD8, and Ki-67 were calculated using the HALO 3.3 software (India Labs, Albuquerque, NM, USA).

### Statistical analysis

All statistical analyses were done with GraphPad Prism 10 software (San Diego, CA, USA). Data are presented as mean ± SEM. *p-*values were obtained using one or two-way analysis of variance (ANOVA) with Tukey’s or Šídák’s multiple comparison test, *F*-test, or two-tailed unpaired *t*-test with 95% confidence interval for evaluation of statistical significance compared with the untreated controls. *p* < 0.05 was considered statistically significant.

## Results

### Addition of ruxolitinib improves UCB-Tregs suppressor function and decreases inflammation of SLE

**Figure 1:**
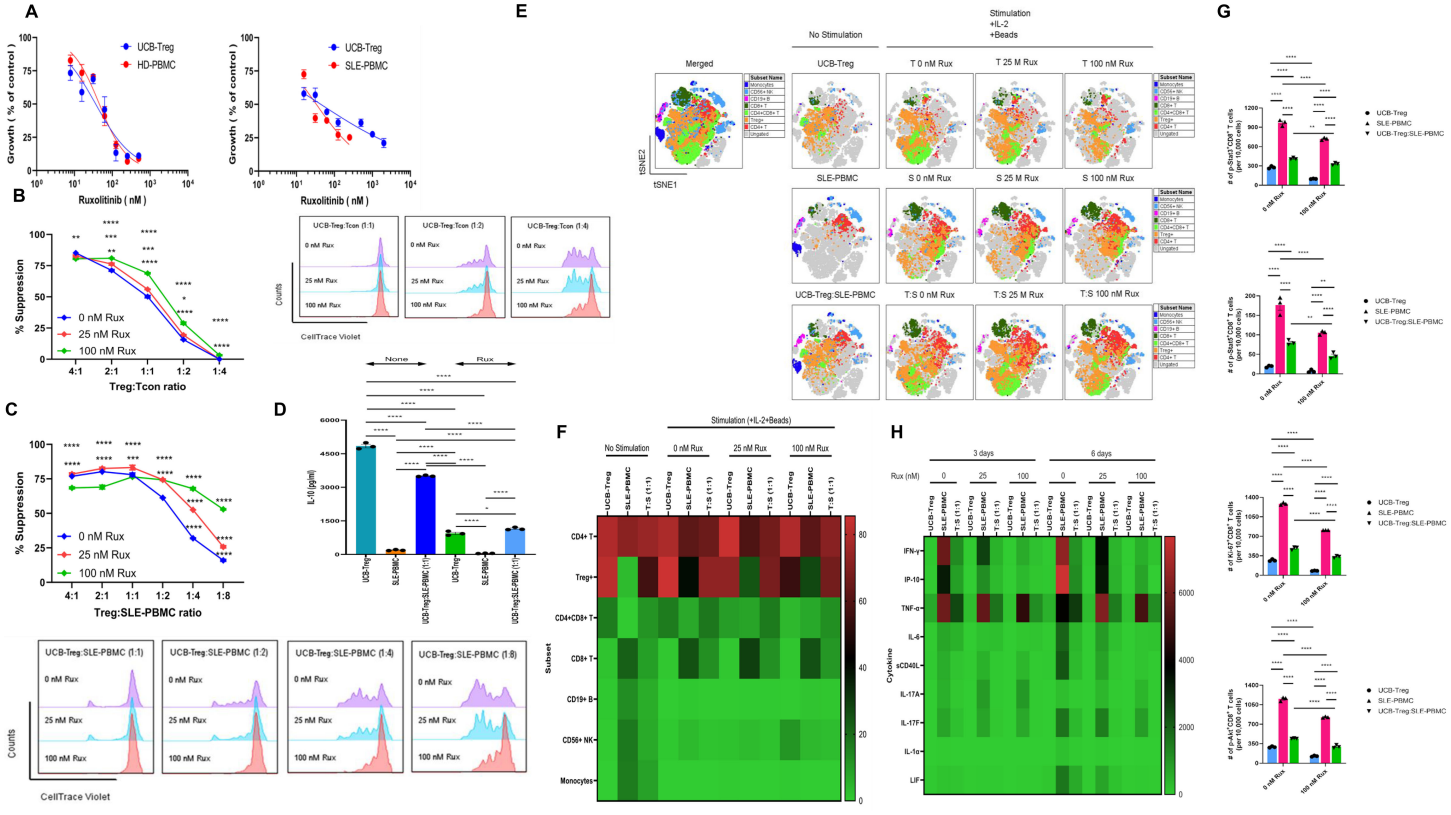
Addition of ruxolitinib synergizes with UCB-Tregs to suppress SLE-PBMCs and decrease inflammatory cytokine secretion. **(A)** Cytotoxicity of ruxolitinib against HD-PBMCs, SLE-PBMCs, or UCB-Tregs when used as a single agent. **(B)** Assessment of UCB-Tregs suppression function on proliferation of Tcons from healthy donor (left panel) and representative histogram (right panel) of suppressive activity of UCB-Tregs against Tcons when cocultured at 1:1, 1:2, and 1:4 ratio at 0, 25, or 100 nM ruxolitinib. **(C)** Assessment of UCB-Tregs suppression function on proliferation of SLE-PBMCs (top panel) in the absence and presence of ruxolitinib and representative histogram (bottom panel) of suppressive activity of UCB-Tregs against SLE-PBMCs when cocultured at 1:1, 1:2, 1:4, or 1:8 ratio at 0, 25, or 100 nM ruxolitinib. **(D)** Production levels of soluble IL-10 in the absence and presence of 25 nM ruxolitinib. Data are presented as mean ± SEM (*n* = 3). *p* < 0.05 was considered statistically significant. *p < 0.05; **p < 0.01; ***p < 0.001; ****p < 0.0001 by one- or two-way ANOVA with Tukey’s multiple comparison tests. **(E)** Subset analysis on the tSNE map. **(F)** Quantification analysis of subsets. CD4^+^ T, CD4^+^CD25^+^CD127^low^ Treg^+^, CD4^+^CD8^+^ T, CD8^+^ T, CD19^+^ B, CD56^+^ NK cells, and CD14^+^ monocytes were quantified. Data are presented as mean ± SEM (*n* = 5). **(G)** Quantification analysis of p-Stat3^+^, p-Stat5^+^, Ki-67^+^, or p-Akt^+^CD8+ T cells. p-Stat3^+^, p-Stat5^+^, Ki-67^+^, or p-Akt^+^CD8+ T cells were quantified. Data are presented as mean ± SEM (*n* = 3). *p-*values were obtained using two-way analysis of variance (ANOVA) with Tukey’s or Šídák’s multiple comparison test and *p* < 0.05 was considered statistically significant. **(H)** Production levels of soluble cytokines on heatmap. UCB-Tregs, SLE-PBMCs, or UCB-Tregs:SLE-PBMC (1:1) cocultures were cultured and the production levels of human soluble cytokines in the 3- or 6-day cell culture supernatants were measured as described in Materials and Methods. Data are presented as mean ± SEM (*n* = 2–3). *p-*values were obtained using two-way analysis and *p* < 0.05 was considered statistically significant.

We have previously demonstrated that CD4^+^CD25^+^127^low^ UCB-Tregs can inhibit various inflammatory cytokines and improve skin damage in a SLE xenograft model (22). Ruxolitinib has been shown to inhibit inflammatory cytokines in cutaneous lupus erythematosus (26). To examine whether addition of ruxolitinib to adoptive therapy with UCB-Tregs can enhance their anti-inflammatory effect in SLE, we first examined the IC_50_ values for ruxolitinib in coculture with UCB-Tregs, HD-PBMCs, or SLE-PBMCs. As shown in **Figure 1A**, no differences were observed in the ruxolitinib IC_50_ among the three different cell types after 6 days of incubation (IC_50_ = 36.3–43.9 nmol/L, 45.5 nmol/L, and 33.4 nmol/L, respectively), suggestive of no direct impact of the pathogenicity of SLE on ruxolitinib effect. Since ruxolitinib has been shown to have differential effect on Tregs *in vivo* (31), we next assessed the impact of the addition of ruxolitinib on UCB-Treg function. The addition of ruxolitinib at 25 or 100 nM synergized with UCB-Tregs to suppress proliferation of HD-Tcon (two-way ANONA interaction: *p* < 0.0001; Treg: Tcon ratio: *p* < 0.0001; Rux conc: *p* < 0.0001) (**Figure 1B**) and SLE-PBMC (two-way ANOVA interaction: *p* < 0.0001; Treg: SLE-PBMC ratio: *p* < 0.0001; Rux conc: *p* < 0.0001) (**Figure 1C**), across different cell ratios, especially as low as the 1:8 ratio of UCB-Treg to SLE-PBMC. We next examined whether ruxolitinib can affect UCB-Tregs’ ability to secrete the suppressor cytokine, IL10 (22). As shown in **Figure 1D**, on day 3 of culture, high levels of IL-10 were detected in the supernatants of UCB-Tregs (4,844 ± 78 pg/mL) whereas the production level of soluble IL-10 was 190 ± 14 pg/mL in SLE-PBMC alone and 3,505 ± 18 pg/mL in the coculture of UCB-Tregs with SLE-PBMC. In the presence of 25 nM ruxolitinib, the respective IL-10 secretion levels in the culture supernatants decreased to 941 ± 53 pg/mL, 46 ± 6 pg/mL, and 1,149 ± 33 pg/mL, respectively (one-way ANOVA, *p* < 0.0001).

To examine the impact of the addition of ruxolitinib to UCB-Tregs on the immune cell compartment of SLE-PBMCs, CD4^+^ T, CD4^+^CD25^+^CD127^low^ Treg^+^, CD4^+^CD8^+^ T, CD8^+^ T, CD19^+^ B, CD56^+^ NK cells, and CD14^+^ monocytes were gated (**Supplementary Figure S1**). As shown in **Figures 1E, F** and **Supplementary Figure S2**, coculture with UCB-Tregs decreased the inflammatory cell subpopulation of SLE-PBMCs. Three-day coculture increased the percentages of CD4^+^CD25^+^CD127^low^ Treg cells and decreased those of CD19^+^ B cells, CD56^+^ NK cells, and CD14^+^ monocytes in UCB-Tregs, SLE-PBMCs, and UCB-Treg:SLE-PBMC, respectively. Interestingly, a significant reduction of CD8^+^ T cells was observed in the UCB-Treg:SLE-PBMC coculture but not SLE-PBMC cultures in the absence and presence of ruxolitinib. The addition of ruxolitinib led to an increase in the double-positive (DP) CD4^+^CD8^+^ T cells in SLE-PBMC+UCB-Tregs coculture.

We next examined whether ruxolitinib can affect STAT3 signaling in SLE-PBMCs and/or UCB-Tregs. A significant reduction of p-STAT3^+^, Ki-67^+^, and p-Akt^+^CD8^+^ T cells was observed in 100 nM ruxolitinib-treated UCB-Tregs and SLE-PBMCs (*p* < 0.0001) and UCB-Treg: SLE-PBMC coculture (*p* < 0.01 for p-Stat3^+^ and *p* < 0.0001 for Ki-67^+^ and p-Akt^+^) (**Figure 1G**). In addition, SLE-PBMCs (*p* < 0.0001) and UCB-Treg: SLE-PBMC coculture (*p* < 0.01) showed a significant reduction of p-STAT5^+^CD8^+^ T cells after treatment with 100 nM ruxolitinib. As shown in **Figure 1H**, the addition of ruxolitinib and/or UCB-Tregs to SLE-PBMCs significantly decreased the soluble form of inflammatory cytokines, including IFN-γ, IP-10, TNF-α, IL-6, sCD40L, IL-17A, IL-17F, IL-1α, and LIF (*p* < 0.0001). Interestingly, ruxolitinib did not have any effect on TNF-α production and surprisingly seemed to increase soluble IL-17A production by SLE-PBMCs in a dose-dependent manner whereas UCB-Tregs led to a significant reduction of IL-17A.

### The addition of ruxolitinib to UCB-Tregs improves their persistence *in vivo* and cutaneous lesions of SLE

**Figure 2:**
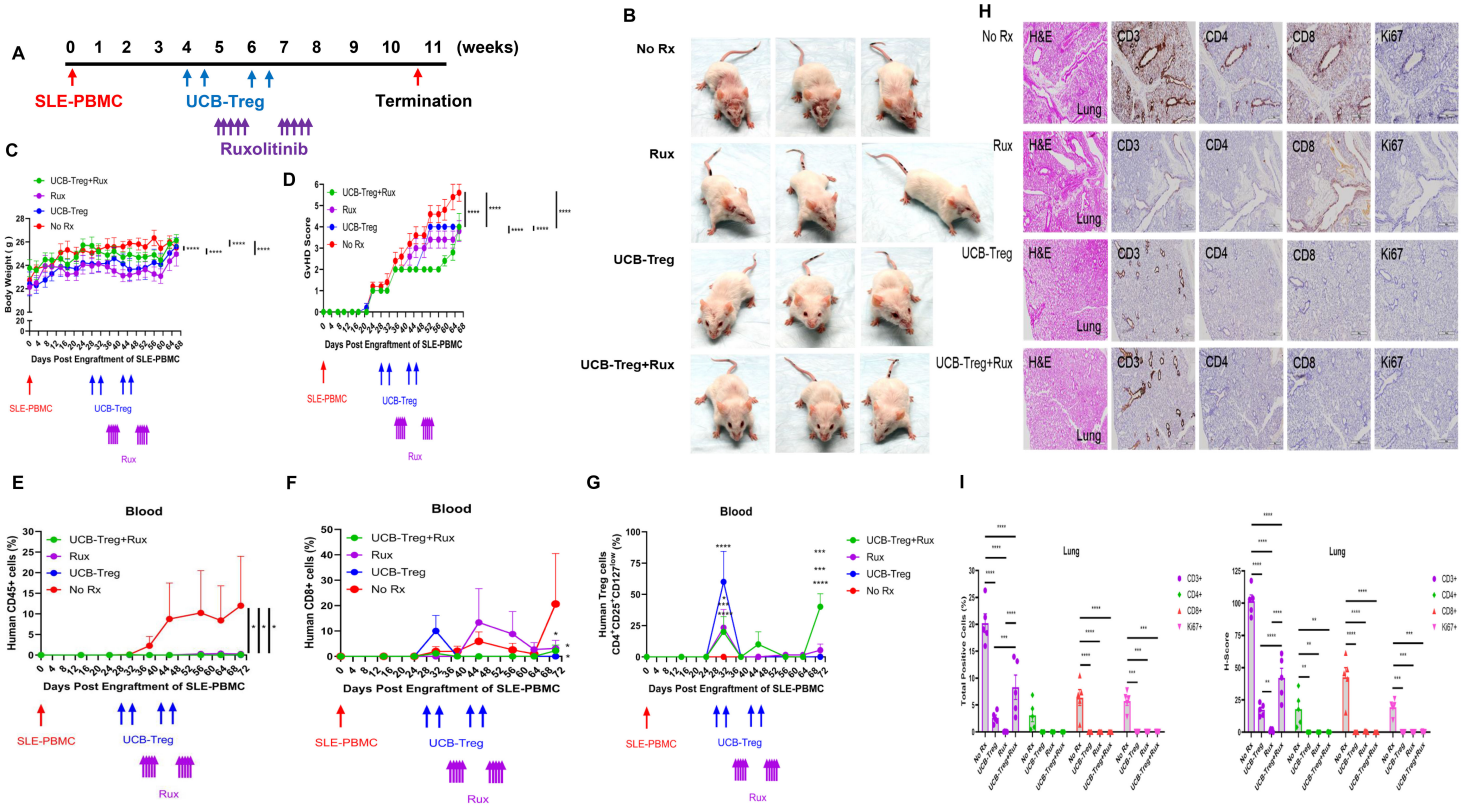
The addition of ruxolitinib increases UCB-Tregs persistence *in vivo* and resolves inflammation. **(A)** Schematic summary of multiple therapy for SLE treatment in a SLE xenograft model. *In vivo* efficacy assessment was done using a SLE xenograft model as described previously (1, 7). Mice were divided into four groups (*n* = 5 mice/group) after they displayed human immune cells. On day 28, day 32, day 42, and day 46, 5 × 10^6^ UCB-Tregs were infused into SLE xenografts intravenously for treatment. Ninety micrograms of ruxolitinib per day was dosed orally on days 35, 36, 37, 38, 39, 49, 50, 51, 52, and 53. **(B)** Photograph of representative mouse from the No Rx, Rux, UCB-Treg, and UCB-Treg+Rux group exhibiting skin changes. Longitudinal monitoring of **(C)** body weight and **(D)** GVHD score in SLE xenografts. Body weight and GvHD score were monitored twice per week or weekly. **(E)** Sustained reduction in circulating human CD45^+^ cells in UCB-Treg, ruxolitinib, and UCB-Treg+ruxolitinib recipients. **(F)** Reduction in circulating human CD8^+^ T cells in all treatment recipients. **(G)** Sustained increase in circulating human CD4^+^CD25^+^CD127^low^ Treg cells in UCB-Treg+ruxolitinib recipients. Data are presented as mean ± SEM (*n* = 5). *p* < 0.05 was considered statistically significant. *p < 0.05; ***p < 0.001; ****p < 0.0001 by two-way ANOVA with Tukey’s multiple comparison tests or Student *t-*test. **(H)** UCB-Tregs in combination with ruxolitinib improve lung tissue damage in SLE xenografts. **(I)** Quantification analysis of total positive cells and *H*-scores for human CD3, CD4, CD8, and Ki-67 in lung tissues. Data are presented as mean ± SEM (*n* = 5). *p* < 0.05 was considered statistically significant. *p < 0.05; **p < 0.01; ***p < 0.001; ****p < 0.0001 by two-way ANOVA with Tukey’s multiple comparison tests.

Using the SLE xenograft model as described previously (22), 4 weeks were allowed for human disease to be established in immune-deficient mice followed by multiple treatments of UCB-Tregs (5 × 10^6^ cells) alone, ruxolitinib alone (90 ×g), or a combination of UCB-Tregs and ruxolitinib. UCB-Tregs were administered by tail vein on days 28, 32, 42, and 46. Ruxolitinib was administered by oral gavage on days 35, 36, 37, 38, 39, 49, 50, 51, 52, and 53. UCB-Tregs plus ruxolitinib were administered at their corresponding time points (**Figure 2A**). SLE disease phenotype was evident in the control arm, where mice developed malar, discoid, and erythematous skin rash, and/or hair loss, similar to that observed in human disease (22). The mice in ruxolitinib alone arm but not UCB-Treg recipients with or without ruxolitinib developed noticeable hair loss (**Figure 2B**). The burden of SLE disease was measured by monitoring the change of body weight and GVHD score, longitudinally. An increase in body weight measured over 68 days was observed in all groups (**Figure 2C**, two-way ANOVA Time and Group; *p* < 0.0001). The control SLE group (No Rx) had the highest GVHD score (**Figure 2D**, two-way ANOVA interaction, Time and Group; *p* < 0.0001), circulating human CD45^+^ cells (**Figure 2E**, *p* < 0.05), and circulating human CD8^+^ T cells (**Figure 2F**, *p* < 0.05), when compared to either of the treatment arms (Rux, UCB-Treg, and UCB-Treg+Rux). The percentage of circulating human CD4^+^CD25^high^CD127^low^ Treg cells was significantly increased following UCB-Tregs injection and was detected up to day +70 in the UCB-Treg+ruxolitinib recipients (**Figure 2G**, *p* < 0.0001).

As shown in **Figure 2H**, lung tissue in the control arm (No Rx) and in the ruxolitinib recipients (Rux) showed significant alveolar disruption whereas UCB-Treg and UCB-Treg+ruxolitinib recipients preserved their alveolar air space. Compared with the control arm (No Rx), lung tissue in all treatment groups had a significant reduction in the total positive cells and the *H*-score for human CD3, CD4, CD8, and Ki-67 (**Figure 2I**, *p* < 0.0001 for Group and Human Markers). All treatment groups have well-preserved spleen tissue architecture whereas the control arm (No Rx) showed lymphocytic infiltration into red and white pulp with splenomegaly (**Supplementary Figure S3A**). Compared with the control group (No Rx), all treatment groups had a significant reduction in the total positive cells and the *H*-score for human CD3, CD4, CD8, and Ki-67 (**Supplementary Figure S3B**, *p* < 0.0001 for Group and Human Markers).

### The addition of ruxolitinib to UCB-Tregs reduces systemic inflammation without added benefits for reduction in albuminuria and anti-dsDNA IgG Ab

**Figure 3:**
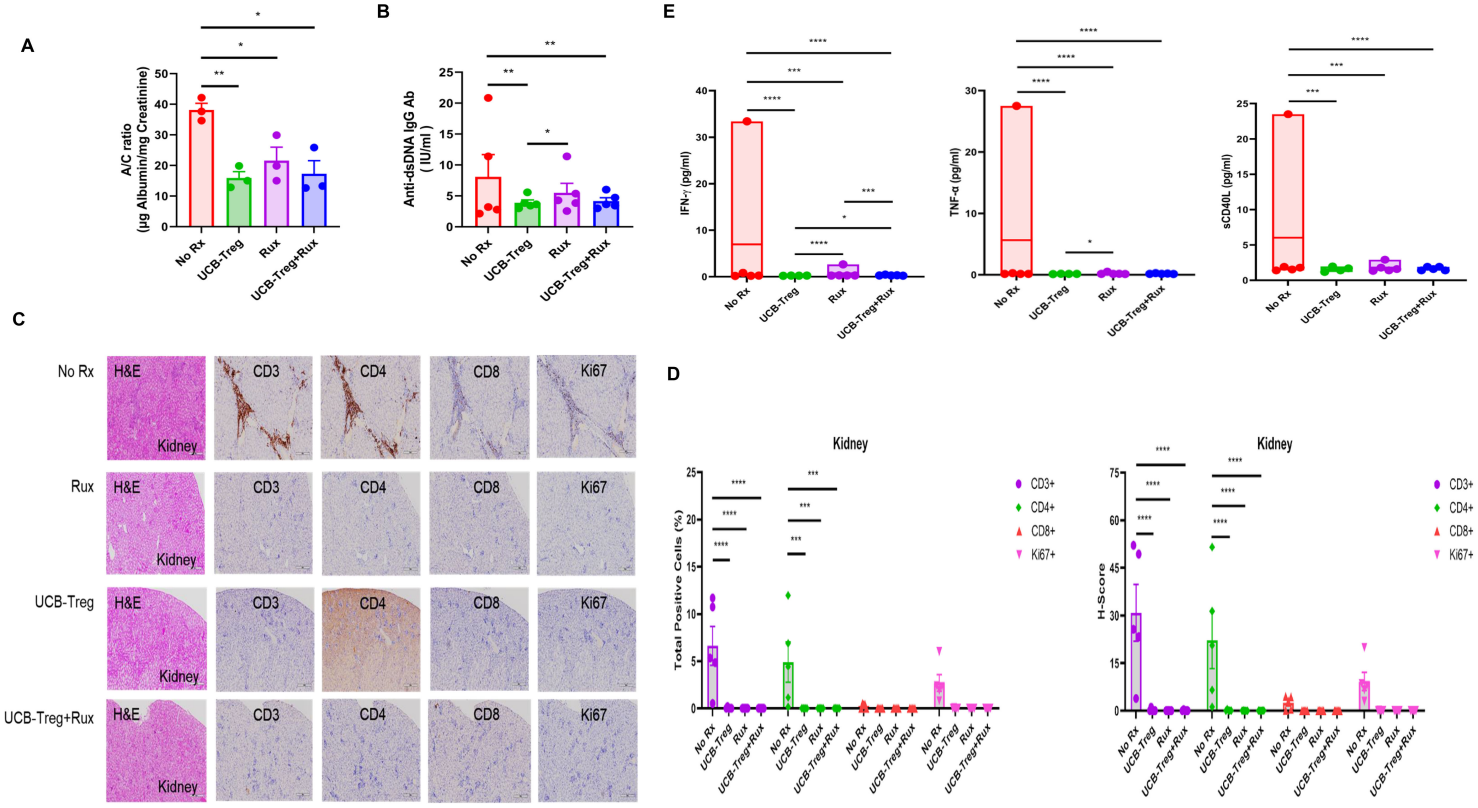
The addition of ruxolitinib does not add benefits to UCB-Tregs-mediated improvement in albuminuria and anti-dsDNA IgG Ab of SLE. **(A)** Decrease in A/C ratio in SLE xenografts. **(B)** Decrease in anti-dsDNA IgG Ab in SLE xenografts. **(C)** Impact of UCB-Tregs in combination with ruxolitinib on renal tissue in SLE xenografts. **(D)** Quantification analysis of total positive cells and *H*-scores for human CD3, CD4, CD8, and Ki-67 in kidney tissues of SLE xenografts. **(E)** Inhibition of inflammatory cytokine production in all treatment recipients. Data are presented as mean ± SEM (*n* = 3–5). *p* < 0.05 was considered statistically significant. *p < 0.05; **p < 0.01; ***p < 0.001; ****p < 0.0001 by two-tailed unpaired *t-*test or *F*-test.

We have previously shown that UCB-Tregs can treat SLE and decrease albuminuria and anti-dsDNA IgG Ab (22). Here, we examined the impact of adding ruxolitinib to UCB-Treg recipients. As shown in **Figure 3A**, a significant decrease in albuminuria (A/C ratio) was observed in all treatment groups but not the control arm (No Rx vs. Rux or UCB-Treg+Rux, *p* < 0.05; No Rx vs. UCB-Treg, *p* < 0.01). A corresponding decrease in the anti-dsDNA IgG Ab was observed in all treatment arms when compared to control. (**Figure 3B**; control vs. UCB-Treg or UCB-Treg+ruxolitinib, *p* < 0.01; for UCB-Treg vs. Rux, *p* < 0.05). Specifically, no differences were seen between UCB-Treg vs. the UCB-Tregs+Rux arm. We observed that a significant reduction of human CD20^+^ B cells was observed in the spleen of treatment arms when compared to control (**Supplementary Figure S3C**; control vs. Rux, *p* < 0.05; control vs. UCB-Treg+ruxolitinib, *p* < 0.01).

Histopathological analysis of kidney tissue showed significant lymphatic infiltrate in the control arm (No Rx) compared with preservation of the kidney architecture in UCB-Treg and UCB-Treg+ruxolitinib recipients (**Figure 3C**). A significant decrease in the total number (**Figure 3D**, left panel, *p* < 0.0001 for Group and *p* = 0.0288 for Human Marker) of human CD3^+^, CD4^+^, and Ki-67^+^ cells and the *H*-score (**Figure 3D**, right panel, *p* < 0.0001 for Group and *p* = 0.0131 for Human Marker) was observed in all treatment groups but not in the control arm. In addition, all treatment arms showed an improvement at the kidney tissue and functional level and a decrease in systemic inflammation and the expression levels of circulating human inflammatory cytokines including IFN-γ, TNF-α, and sCD40L, which overlapped with those impacted in *in vitro* studies (**Figure 3E**; *p < 0.05, ***p < 0.001, ****p < 0.0001).

## Discussion

Here, we show that the addition of ruxolitinib improves UCB-Tregs’ ability to suppress proliferation of healthy as well as SLE-derived PBMCs. Such synergy translates into prolonged persistence of Tregs *in vivo* and improvement in the skin lesions. Interestingly, we observed that the addition of ruxolitinib decreases Treg cell population within SLE-PBMCs and decreases IL-10 secreted by UCB-Tregs. Such a discrepancy might be explained by the differential effect of ruxolitinib on Tregs based on an underlying disease biology that has been previously described in bone marrow failure, where ruxolitinib increased splenic Tregs in diseased mouse but not in the healthy control (31). A decrease in the distribution of cell populations including T-cell compartment consisting of the CD4 and CD8 subsets as well as Tregs, B-cell compartment, NK cells and monocytes in response to UCB-Tregs, ruxolitinib, and their combination was also observed in our study, which is similar to the reported decrease in the frequencies of splenic CD4^+^ T, CD8^+^ T, and NK-T cells with a significant augmentation of splenic Tregs in a mouse model of autoimmune cholangitis after treatment with ruxolitinib (32). Additionally, hemophagocytic lymphohistiocytosis, a fatal complication of SLE, primarily driven by uncontrolled activation of pathogenic CD8^+^ T cells, has been shown to be responsive to ruxolitinib in a case report (33).

The negative impact of ruxolitinib on the IL-10 secretion from UCB-Tregs was an interesting observation that might be a function of the decrease in the Treg cell number. In contrast, we show that the addition of ruxolitinib in fact increased the cell suppressor function of UCB-Tregs *in vitro* and increased their persistence *in vivo*. Such ruxolitinib-mediated synergy on UCB-Treg function and survival is suggestive of possible additional mechanisms at play, beyond IL-10 secretion. We show that ruxolitinib-mediated suppression of the STAT3/5-AKT pathway in the CD8^+^ T cells in SLE-PBMCs in the presence or absence of UCB-Tregs might suggest an independent mechanism of targeting SLE pathogenesis, since inhibition of STAT3 in T cells in lupus has been shown to delay the onset of nephritis (34). Furthermore, a decrease in the release of multiple inflammatory cytokines, *in vitro* and *in vivo*, including IFN-γ, IP-10, IL-6, sCD40L, IL-17F, IL-1α, and LIF, similar to that shown by ruxolitinib in GVHD (35) and COVID-19 (36), also supports the ruxolitinib-mediated mechanism of synergizing with UCB-Tregs independent of IL-10 secretion in SLE. Specifically, IFN-γ has been shown to be complicit in SLE pathogenesis (37, 38). JAK inhibitors can suppress the IFN signaling in human dendritic cells, reduce CD80/CD86 expression and T-cell stimulation ability (39), and reduce the production of various inflammatory cytokines including IFN-γ (40) in SLE mice. On the other hand, UCB-Tregs, as a single agent, have also been shown to resolve SLE inflammation and decrease inflammatory cytokines including IFN-γ, IP-10, TNF-α, IL-6, IL-17A, sCD40L, and IL-1α (22).

Furthermore, a unilateral decrease in pathogenic CD8^+^ T cells in circulation and in tissues underscores the synergistic impact of adding ruxolitinib to enhance UCB-Tregs ability to suppress SLE-PBMC (22). In SLE, pathogenic IL-17-producing CD4CD8 double-negative (DN) T cells are thought to originate from autoreactive CD8^+^ T cells, which also contribute to the induction and maintenance of systemic autoimmunity (41, 42). Specifically, these DN T cells promote autoantibody production; essentially, they are considered a key pathogenic cell population in SLE (43). In our study, we observed that UCB-Treg cells increased CD4/CD8 double-negative T cells after treatment with 100 nM ruxolitinib; however, such an increase was not associated with an increase in the production of IL-17A and IL-17F. In fact, the coculture of UCB-Tregs with SLE-PBMC significantly decreased the production of IL-17A and IL-17F by SLE-PBMCs (**Figure 1H**). On the other hand, the CD4CD8 double positive (DP) T cell population was shown to have a suppressive effect on the production of autoantibodies including antinuclear antibody and anti-dsDNA Ab in SLE (44) and is increased in response to UCB-Tregs in a SLE xenogeneic model (22). Although their exact significance is unclear, these DP T cells may represent a transition of T-cell population towards further differentiation (45). *In vivo* terminally differentiated effector CD4^+^ T cells may acquire the alpha-chain of CD8^+^ T cells, and these CD4^+^CD8^+^ T cells have been identified in autoimmune and chronic inflammatory disorders (46). Another study shows no difference in the DP T-cell population in SLE and healthy controls (47). Future studies would be required to better understand the impact of this subpopulation of T cells in SLE.

It is not surprising that the addition of ruxolitinib to UCB-Tregs did not add to their existing benefit of controlling systemic renal disease manifestations, as measured by urine albumin/creatinine ratio as well as anti-dsDNA IgG Ab. We have previously shown that single-agent UCB-Tregs can decrease anti-dsDNA IgG antibody and improve renal function in SLE (22). Therefore, the maximum benefit of adding ruxolitinib might be for control of dermal manifestation as seen in our data as well as reported by others (25–27).

Taken together, our results demonstrate that the addition of ruxolitinib to UCB-Tregs augments the inhibition of pathogenic CD8^+^ T cells expressing p-STAT3^+^, p-STAT5^+^, and p-Akt^+^, and the reduction of soluble IFN-γ, IP-10, TNF-α, IL-6, sCD40L, IL-17A, IL-17F, IL-1α, and LIF production, and ruxolitinib improves UCB-Treg cell persistence and decreases CD3^+^, CD4^+^ T, CD8^+^ T, and Ki-67^+^ cell tissue infiltration but has no added benefits to albuminuria and anti-dsDNA IgG Ab. In conclusion, the addition of ruxolitinib to UCB-Tregs in SLE is a viable therapeutic strategy and should be explored in the clinical setting, especially for dermal manifestations.

## Data Availability

The raw data supporting the conclusions of this article will be made available by the authors, without undue reservation.

## Supplementary material

Supplementary Figure 1 Representative FACS analysis of subsets. The percentage of CD4^+^ T, CD4^+^CD25^+^CD127^low^ Treg^+^, CD4^+^CD8^+^ T, CD8^+^ T, CD19^+^ B, CD56^+^ NK cells, and CD14^+^ monocytes were acquired on a BD LSRFortessa X-20 flow cytometer and analyzed using FlowJo software.

Supplementary Figure 2 Quantification analysis of CD4^+^ T, CD4^+^CD25^+^CD127^low^ Treg^+^, CD4^+^CD8^+^ T, CD8^+^ T, CD19^+^ B, CD56^+^ NK cells, and CD14^+^ monocytes were quantified. Data are presented as mean ± SEM (n=3). *P*<0.05 was considered statistically significant. *P*<0.05 was considered statistically significant. **P*<0.05; ****P*< 0.001; *****P*<0.0001 by Student *t-*test.

Supplementary Figure 3 Addition of Ruxolitinib improves spleen damage in SLE xenografts. **(A)** Impact of UCB-Tregs in combination with ruxolitinib on spleen tissue in SLE xenografts. **(B)** Quantification analysis of total positive cells and H-scores for human CD3, CD4, CD8, and Ki-67 in spleen tissues. Data are presented as mean ± SEM (n=5). *P*<0.05 was considered statistically significant. **P*<0.05; *****P*<0.0001 by two-way ANOVA with Tukey’s multiple comparison tests. **(C)** Quantification analysis of human CD20+ B cells in spleen tissues. Data are presented as mean ± SEM (n=5). *P*<0.05 was considered statistically significant. **P*<0.05; ***P*<0.01 by Student *t-*test.

## References

[1] Paredes JL Fernandez-Ruiz R Niewold TB. T cells in systemic lupus erythematosus. Rheum Dis Clin North Am. (2021) 47:379–93. doi: 10.1016/j.rdc.2021.04.005 PMC8262037 34215369

[2] Suarez-Fueyo A Bradley SJ Tsokos GC. T cells in systemic lupus erythematosus. Curr Opin Immunol. (2016) 43:32–8. doi: 10.1016/j.coi.2016.09.001 PMC5125867 27636649

[3] Katsuyama T Tsokos GC Moulton VR. Aberrant T cell signaling and subsets in systemic lupus erythematosus. Front Immunol. (2018) 9:1088. doi: 10.3389/fimmu.2018.01088 29868033 PMC5967272

[4] Lee SK Silva DG Martin JL Pratama A Hu X Chang P-P. Interferon-gamma excess leads to pathogenic accumulation of follicular helper T cells and germinal centers. Immunity. (2012) 37:880–92. doi: 10.1016/j.immuni.2012.10.010 23159227

[5] Shah D Kiran R Wanchu A Bhatnagar A. Oxidative stress in systemic lupus erythematosus: relationship to Th1 cytokine and disease activity. Immunol Lett. (2010) 129:7–12. doi: 10.1016/j.imlet.2010.01.005 20105444

[6] Raphael I Nalawade S Eagar TN Forsthuber TG. T cell subsets and their signature cytokines in autoimmune and inflammatory diseases. Cytokine. (2015) 74:5–17. doi: 10.1016/j.cyto.2014.09.011 25458968 PMC4416069

[7] Nakajima A Hirose S Yagita H Okumura K. Roles of IL-4 and IL-12 in the development of lupus in NZB/W F1 mice. J Immunol. (1997) 158:1466–72. doi: 10.4049/jimmunol.158.3.1466 9013993

[8] Zickert A Amoudruz P Sundström Y Rönnelid J Malmström V Gunnarsson I. IL-17 and IL-23 in lupus nephritis - association to histopathology and response to treatment. BMC Immunol. (2015) 16:7. doi: 10.1186/s12865-015-0070-7 25887118 PMC4326189

[9] Dai H He F Tsokos GC Kyttaris VC. IL-23 limits the production of IL-2 and promotes autoimmunity in lupus. J Immunol. (2017) 199:903–10. doi: 10.4049/jimmunol.1700418 PMC5526729 28646040

[10] Mizui M Tsokos GC. Targeting regulatory T cells to treat patients with systemic lupus erythematosus. Front Immunol. (2018) 9:786. doi: 10.3389/fimmu.2018.00786 29755456 PMC5932391

[11] Sakaguchi S. Naturally arising Foxp3-expressing CD25+CD4+ regulatory T cells in immunological tolerance to self and non-self. Nat Immunol. (2005) 6:345–52. doi: 10.1038/ni1178 15785760

[12] Josefowicz SZ Lu LF Rudensky AY. Regulatory T cells: mechanisms of differentiation and function. Annu Rev Immunol. (2012) 30:531–64. doi: 10.1146/annurev.immunol.25.022106.141623 PMC6066374 22224781

[13] Sakaguchi S Wing K Onishi Y Prieto-Martin P Yamaguchi T. Regulatory T cells: how do they suppress immune responses? Int Immunol. (2009) 21(10):1105–11. doi: 10.1093/intimm/dxp095 19737784

[14] Akkaya B Shevach EM. Regulatory T cells: Master thieves of the immune system. Cell Immunol. (2020) 355:104160. doi: 10.1016/j.cellimm.2020.104160 32711171 PMC9761694

[15] Dikiy S Rudensky AY. Principles of regulatory T cell function. Immunity. (2023) 56:240–55. doi: 10.1016/j.immuni.2023.01.004 36792571

[16] Mellor-Pita S Citores MJ Castejon R Tutor-Ureta P Yebra-Bango M Andreu JL. Decrease of regulatory T cells in patients with systemic lupus erythematosus. Ann Rheum Dis. (2006) 65:553–4. doi: 10.1136/ard.2005.044974 PMC1798083 16531555

[17] Pan X Yuan X Zheng Y Wang W Shan J Lin F. Increased CD45RA+ FoxP3(low) regulatory T cells with impaired suppressive function in patients with systemic lupus erythematosus. PloS One. (2012) 7:e34662. doi: 10.1371/journal.pone.0034662 22506043 PMC3323568

[18] Valencia X Yarboro C Illei G Lipsky PE. Deficient CD4+CD25high T regulatory cell function in patients with active systemic lupus erythematosus. J Immunol. (2007) 178:2579–88. doi: 10.4049/jimmunol.178.4.2579 17277168

[19] Bonelli M Savitskaya A von Dalwigk K Steiner CW Aletaha D Smolen JS. Quantitative and qualitative deficiencies of regulatory T cells in patients with systemic lupus erythematosus (SLE). Int Immunol. (2008) 20:861–8. doi: 10.1093/intimm/dxn044 18469329

[20] Venigalla RKC Tretter T Krienke S Max R Eckstein V Blank N. Reduced CD4+,CD25- T cell sensitivity to the suppressive function of CD4+,CD25high,CD127 -/low regulatory T cells in patients with active systemic lupus erythematosus. Arthritis Rheum. (2008) 58:2120–30. doi: 10.1002/art.23556 18576316

[21] Lyu MA Huang M Zeng K Li L Khoury JD Nishimoto M. Allogeneic cord blood regulatory T cells can resolve lung inflammation. Cytotherapy. (2023) 25:245–53. doi: 10.1016/j.jcyt.2022.10.009 PMC12481963 36437190

[22] Lyu MA Tang X Khoury JD Raso MG Huang M Zeng K. Allogeneic cord blood regulatory T cells decrease dsDNA antibody and improve albuminuria in systemic lupus erythematosus. Front Immunol. (2023) 14:1217121. doi: 10.3389/fimmu.2023.1217121 37736101 PMC10509479

[23] de la Varga Martinez R Rodríguez-Bayona B Añez GA Varo FM Pérez Venegas JJ Brieva JA. Clinical relevance of circulating anti-ENA and anti-dsDNA secreting cells from SLE patients and their dependence on STAT-3 activation. Eur J Immunol. (2017) 47:1211–9. doi: 10.1002/eji.201646872 28463395

[24] Zeiser R von Bubnoff N Butler J Mohty M Niederwieser D Or R. Ruxolitinib for glucocorticoid-refractory acute graft-versus-host disease. N Engl J Med. (2020) 382:1800–10. doi: 10.1056/NEJMoa1917635 32320566

[25] Wenzel J von Holt N Maier J Vonnahme M Bieber T Wolf D. JAK1/2 inhibitor ruxolitinib controls a case of chilblain lupus erythematosus. J Invest Dermatol. (2016) 136:1281–3. doi: 10.1016/j.jid.2016.02.015 26916391

[26] Klaeschen AS Wolf D Brossart P Bieber T Wenzel J. JAK inhibitor ruxolitinib inhibits the expression of cytokines characteristic of cutaneous lupus erythematosus. Exp Dermatol. (2017) 26:728–30. doi: 10.1111/exd.2017.26.issue-8 27892610

[27] Chan ES Herlitz LC Jabbari A. Ruxolitinib attenuates cutaneous lupus development in a mouse lupus model. J Invest Dermatol. (2015) 135:1912–5. doi: 10.1038/jid.2015.107 25789705

[28] Rodriguez-Gil A Escamilla-Gómez V Nufer M Andújar-Sánchez F Lopes-Ramos T Bejarano-García JA. Combined treatment of graft versus host disease using donor regulatory T cells and ruxolitinib. Sci Rep. (2022) 12:8348. doi: 10.1038/s41598-022-12407-x 35589917 PMC9120462

[29] Zeng K Ma H Huang M Lyu MA Sadeghi T Flowers CR. Cord blood T regulatory cells synergize with ruxolitinib to improve GVHD outcomes. Front Transplant. (2024) 3. doi: 10.3389/frtra.2024.1448650 PMC11668690 39722683

[30] Lyu MA Cheung LH Hittelman WN Marks JW Aguiar RCT Rosenblum MG. The rGel/BLyS fusion toxin specifically targets Malignant B cells expressing the BLyS receptors BAFF-R, TACI, and BCMA. Mol Cancer Ther. (2007) 6:460–70. doi: 10.1158/1535-7163.MCT-06-0254 17267661

[31] Aggarwal N Manley AL Chen J Groarke EM Feng X Young NS. Effects of ruxolitinib on murine regulatory T cells are immune-context dependent. Exp Hematol. (2023) 125-126:16–9. doi: 10.1016/j.exphem.2023.07.004 PMC10528974 37468118

[32] Shao T Leung PSC Zhang W Tsuneyama K Ridgway WM Young HA. Treatment with a JAK1/2 inhibitor ameliorates murine autoimmune cholangitis induced by IFN overexpression. Cell Mol Immunol. (2022) 19:1130–40. doi: 10.1038/s41423-022-00904-y PMC9508183 36042351

[33] Jung JI Kim JY Kim MH Park JK Lee EY Lee EB. Successful treatment of hemophagocytic lymphohistiocytosis in a patient with systemic lupus erythematosus with ruxolitinib: a case report. J Rheum Dis. (2024) 31:125–9. doi: 10.4078/jrd.2023.0027 PMC10973350 38559795

[34] Yoshida N He F Kyttaris VC. T cell-specific STAT3 deficiency abrogates lupus nephritis. Lupus. (2019) 28:1468–72. doi: 10.1177/0961203319877242 PMC6791775 31551033

[35] Verstovsek S Kantarjian HK Mesa RA Pardanani AD Cortes-Franco J Thomas DA. Safety and efficacy of INCB018424, a JAK1 and JAK2 inhibitor, in myelofibrosis. N Engl J Med. (2010) 363:1117–27. doi: 10.1056/NEJMoa1002028 PMC5187954 20843246

[36] Yeleswaram S Smith P Burn T Covington M Juvekar A Li Y. Inhibition of cytokine signaling by ruxolitinib and implications for COVID-19 treatment. Clin Immunol. (2020) 218:108517. doi: 10.1016/j.clim.2020.108517 32585295 PMC7308779

[37] Liu W Li M Wang Z Wang J. IFN-gamma mediates the development of systemic lupus erythematosus. BioMed Res Int. (2020) 2020:7176515. doi: 10.1155/2020/7176515 33123584 PMC7586164

[38] Liu W Zhang S Wang J. IFN-gamma, should not be ignored in SLE. Front Immunol. (2022) 13:954706. doi: 10.3389/fimmu.2022.954706 36032079 PMC9399831

[39] Kubo S Yamaoka K Kondo M Yamagata K Zhao J Iwata S. The JAK inhibitor, tofacitinib, reduces the T cell stimulatory capacity of human monocyte-derived dendritic cells. Ann Rheum Dis. (2014) 73:2192–8. doi: 10.1136/annrheumdis-2013-203756 24013646

[40] Lu LD Stump KL Wallace NH Dobrzanski P Serdikoff C Gingrich DE. Depletion of autoreactive plasma cells and treatment of lupus nephritis in mice using CEP-33779, a novel, orally active, selective inhibitor of JAK2. J Immunol. (2011) 187:3840–53. doi: 10.4049/jimmunol.1101228 21880982

[41] Chen PM Tsokos GC. T cell abnormalities in the pathogenesis of systemic lupus erythematosus: an update. Curr Rheumatol Rep. (2021) 23:12. doi: 10.1007/s11926-020-00978-5 33512577 PMC8601587

[42] Chen PM Tsokos GC. The role of CD8+ T-cell systemic lupus erythematosus pathogenesis: an update. Curr Opin Rheumatol. (2021) 33:586–91. doi: 10.1097/BOR.0000000000000815 PMC8567317 34183542

[43] Crispin JC Oukka M Bayliss G Cohen RA Van Beck CA Stillman IE. Expanded double negative T cells in patients with systemic lupus erythematosus produce IL-17 and infiltrate the kidneys. J Immunol. (2008) 181:8761–6. doi: 10.4049/jimmunol.181.12.8761 PMC2596652 19050297

[44] Wu Y Cai B Feng W Yang B Huang Z Zuo C. Double positive CD4+CD8+ T cells: key suppressive role in the production of autoantibodies in systemic lupus erythematosus. Indian J Med Res. (2014) 140:513–9. PMC4277137 25488445

[45] Nunes-Cabaco H Caramalho I Sepúlveda N Sousa AE. Differentiation of human thymic regulatory T cells at the double positive stage. Eur J Immunol. (2011) 41:3604–14. doi: 10.1002/eji.201141614 21932449

[46] Parel Y Chizzolini C. CD4+ CD8+ double positive (DP) T cells in health and disease. Autoimmun Rev. (2004) 3:215–20. doi: 10.1016/j.autrev.2003.09.001 15110234

[47] Yuan S Zeng Y Li J Wang C Li W He Z. Phenotypical changes and clinical significance of CD4(+)/CD8(+) T cells in SLE. Lupus Sci Med. (2022) 9(1):e000660. doi: 10.1136/lupus-2022-000660 35732344 PMC9226979

