# Supplementary figures and images for: Ruxolitinib synergizes with regulatory T cells to improve inflammation but has no added benefits in decreasing albuminuria in SLE
Source: Front Immunol. 2025 Feb 5;16:1449693. doi: 10.3389/fimmu.2025.1449693 (PMC11836023; doi:10.3389/fimmu.2025.1449693)

Figure S1

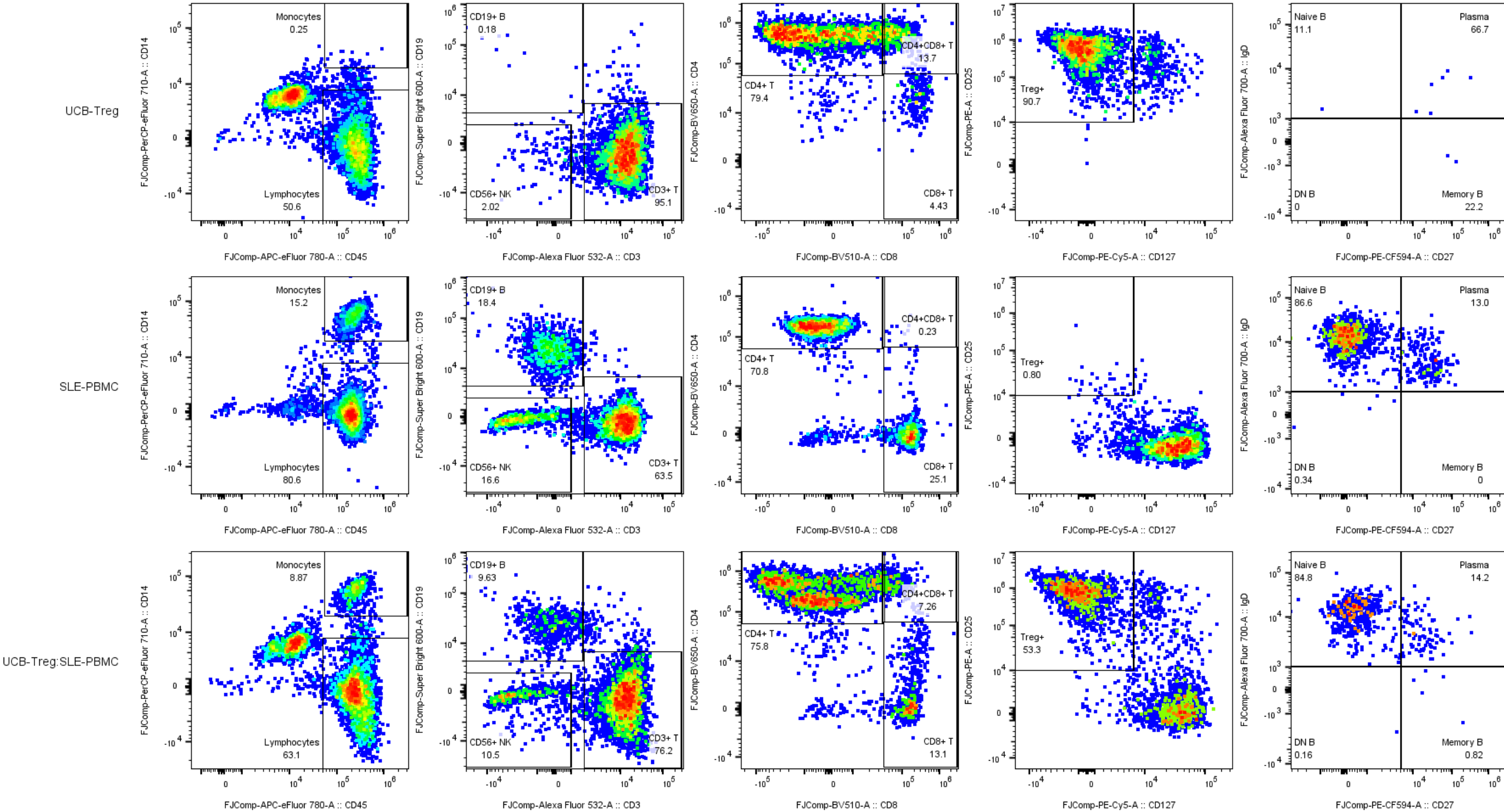

Supplement: Supplementary Figure 1 — Representative FACS analysis of subsets. The percentage of CD4+ T, CD4+CD25+CD127low Treg+, CD4+CD8+ T, CD8+ T, CD19+ B, CD56+ NK cells, and CD14+ monocytes were acquired on a BD LSRFortessa X-20 flow cytometer and analyzed using FlowJo software. [file DataSheet1.pdf]

Figure S2

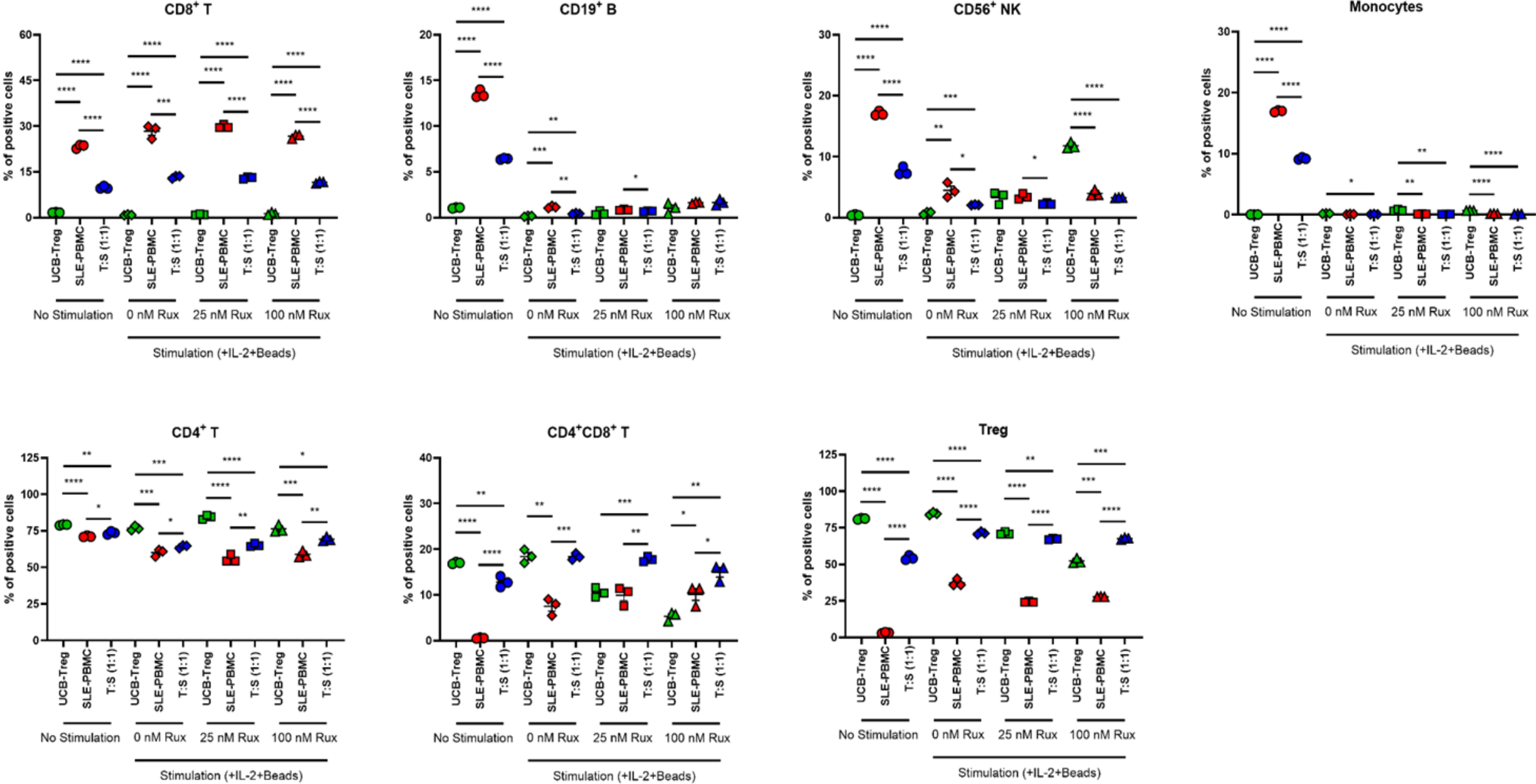

Supplement: Supplementary Figure 2 — Quantification analysis of CD4+ T, CD4+CD25+CD127low Treg+, CD4+CD8+ T, CD8+ T, CD19+ B, CD56+ NK cells, and CD14+ monocytes were quantified. Data are presented as mean ± SEM (n=3). P<0.05 was considered statistically significant. P<0.05 was considered statistically significant. *P<0.05; ***P< 0.001; ****P<0.0001 by Student t-test. [file DataSheet2.pdf]
