# Supplementary material for: Ruxolitinib synergizes with regulatory T cells to improve inflammation but has no added benefits in decreasing albuminuria in SLE
Source: Front Immunol. 2025 Feb 5;16:1449693. doi: 10.3389/fimmu.2025.1449693 (PMC11836023; doi:10.3389/fimmu.2025.1449693)
Supplement: Supplementary Figure 3 — Addition of Ruxolitinib improves spleen damage in SLE xenografts. (A) Impact of UCB-Tregs in combination with ruxolitinib on spleen tissue in SLE xenografts. (B) Quantification analysis of total positive cells and H-scores for human CD3, CD4, CD8, and Ki-67 in spleen tissues. Data are presented as mean ± SEM (n=5). P<0.05 was considered statistically significant. *P<0.05; ****P<0.0001 by two-way ANOVA with Tukey’s multiple comparison tests. (C) Quantification analysis of human CD20+ B cells in spleen tissues. Data are presented as mean ± SEM (n=5). P<0.05 was considered statistically significant. *P<0.05; **P<0.01 by Student t-test. [file DataSheet3.pdf]

Figure S3

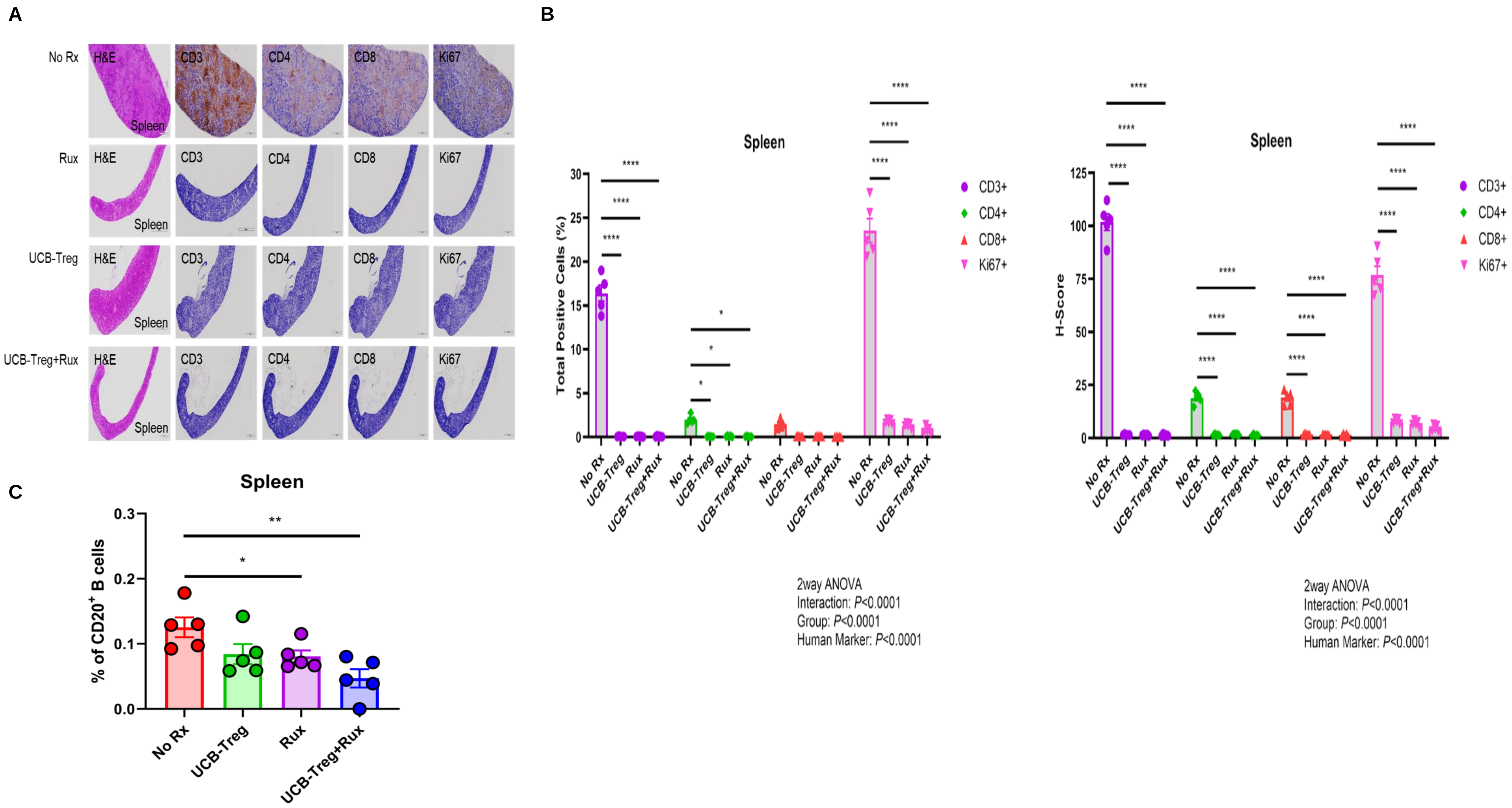
A
No Rx
Rux
UCB-Treg
UCB-Treg+Rux
H&E
CD3
CD4
CD8
Ki67
Spleen
B
Spleen
Total Positive Cells (%)
H-Score
CD3+
CD4+
CD8+
Ki67+
No Rx
UCB-Treg
Rux
UCB-Treg+Rux
2way ANOVA
Interaction: P<0.0001
Group: P<0.0001
Human Marker: P<0.0001
C
Spleen
% of CD20+ B cells
**
*
